# Patient and provider costs of the new BPaL regimen for drug-resistant tuberculosis treatment in South Africa: A cost-effectiveness analysis

**DOI:** 10.1371/journal.pone.0309034

**Published:** 2024-10-21

**Authors:** Denise Evans, Kamban Hirasen, Clive Ramushu, Lawrence Long, Edina Sinanovic, Francesca Conradie, Pauline Howell, Xavier Padanilam, Hannetjie Ferreira, Ebrahim Variaiva, Shakira Rajaram, Aastha Gupta, Sandeep Juneja, Norbert Ndjeka

**Affiliations:** 1 Faculty of Health Sciences, Health Economics and Epidemiology Research Office, University of the Witwatersrand, Johannesburg, South Africa; 2 Faculty of Health Sciences, Health Economics Division, School of Public Health, University of Cape Town, Cape Town, South Africa; 3 Department of Global Health, School of Public Health, Boston University, Boston, MA, United States of America; 4 Faculty of Health Sciences, Clinical HIV Research Unit, University of the Witwatersrand, Johannesburg, South Africa; 5 Sizwe Tropical Disease Hospital, Sandringham, Johannesburg, South Africa; 6 Tshepong Hospital, Jouberton, Klerksdorp, South Africa; 7 TB Alliance, New York, NY, United States of America; 8 South African National Department of Health, Pretoria, South Africa; Kings College Hospital, UNITED KINGDOM OF GREAT BRITAIN AND NORTHERN IRELAND

## Abstract

**Background:**

Drug-resistant (DR) tuberculosis (TB) is typically characterized by resistance to a single or combination of first- and/or second-line anti-TB agents and commonly includes rifampicin-resistant (RR)-TB, multidrug-resistant (MDR)-TB, pre-extensively drug-resistant (pre-XDR)-TB and XDR-TB. Historically, all variations of DR-TB required treatment with second-line drugs which are less effective and more toxic than first-line options, have a longer treatment duration and are more expensive to both patients and providers. The World Health Organization (WHO) now recommends a new second-line 3-drug 6-month all-oral regimen consisting of bedaquiline, pretomanid, and linezolid referred to as BPaL. We estimate patient and provider costs of DR-TB treatment with BPaL compared to the current standard of care in South Africa.

**Methods and findings:**

In coordination with South Africa’s BPaL clinical access programme (CAP) we conducted an economic evaluation of A) patient costs through a cross-sectional patient cost survey and B) provider costs through a bottom-up costing analysis consisting of a retrospective medical record review (patient resource-use) and top-down financial record review (fixed/shared costs such as overhead). Across both costing perspectives, we compare costs of 1) BPaL, to current standard of care options including the 2) 9-11-month standard short oral regimen (SSOR) and 3) 18-21-month standard long oral regimen (SLOR). Eligible patients included those ≥14 years old with confirmed sputum pulmonary RR/MDR-TB, pre-XDR or XDR-TB. All costs are reported in 2022 United States Dollar (US$).

A total of 72 patients were enrolled and completed the patient cost survey (41.7% on BPaL, 16.7% on the SSOR and 41.7% on the SLOR).

Mean on-treatment patient costs were lowest among those on BPaL ($56.6) and increased four-fold among those on the SSOR ($228.1) and SLOR ($224.7). Direct medical patient costs were negligible across all treatment regimens, while direct non-medical patient and guardian costs for travel, food and nutritional supplementation accounted for the largest proportion of total costs ($54.6, $227.8 and $224.3 for BPaL, the SSOR and SLOR respectively).

In assessing provider costs, a total of 112 medical records were reviewed (37.5%, 41.1% and 21.4% on BPaL, the SSOR and SLOR respectively). Total provider costs for producing a favorable treatment outcome (cured/completed treatment) were similar among those on BPaL ($4,948.7 per patient) and the SSOR ($4,905.6 per patient) with costs increasing substantially among those on the SLOR ($8,919.9 per patient). Based on incremental cost-effectiveness ratios (ICERs), at even the lowest willingness to pay (WTP) threshold, treatment with the new BPaL regimen was more cost-effective than current standard of care treatment options (ICER: $311.4 < WTP: $3,341).

**Conclusions:**

When using the newly recommended BPaL regimen, cost to patients decreased by 75% compared to current standard of care treatment options in South Africa. Due in part to higher resource-use within the BPaL CAP offsetting the shorter treatment duration, cost of treatment provision through BPaL and the 9-11-month SSOR were similar. However, when considering cost and treatment outcomes, BPaL was more cost-effective than other standard of care regimens currently available for DR-TB in South Africa.

## Background

Drug-resistant (DR) tuberculosis (TB) is typically characterized by resistance to a single or combination of first- and/or second-line anti-TB agents [1]. Commonly, this includes rifampicin resistant (RR)-TB, multidrug-resistant (MDR)-TB (resistance to rifampicin and isoniazid) [2], pre- extensively-drug resistant (XDR)-TB (RR/MDR-TB with resistance to any fluoroquinolone) as well as XDR-TB (RR/MDR-TB with resistance to any fluoroquinolone and at least bedaquiline and/or linezolid) [3].

According to the World Health Organization (WHO), DR-TB remains a significant global public health challenge, with an estimated 465,000 new cases of RR/MDR-TB reported globally in 2019 [4]. South Africa remains among the seven countries with the highest incidence of RR/MDR-TB reporting over 10,000 new cases in 2021 [5].

Historically, when compared to drug-susceptible (DS)-TB, all variations of DR-TB have required treatment with second-line therapies, which involve more toxic treatment regimens [6]. This necessitates closer monitoring, longer treatment durations, and often results in a higher frequency and intensity of adverse reactions [7], increased hospitalization rates, and poorer treatment outcomes [8]–ultimately increasing the cost of treatment to both patients and providers alike [9].

A promising alternative to traditional second-line therapies is the new 3-drug 6-month all-oral regimen consisting of bedaquiline, pretomanid, and linezolid, hereafter referred to as BPaL.Evidence in support of this regimen has come largely from the Nix-TB [2] and ZeNix [10] clinical trials conducted in South Africa which demonstrated 90% treatment success rates six months post-treatment initiation among patients resistant to rifampicin and to either a fluoroquinolone and/or an aminoglycoside, with both trials reporting significantly higher success rates compared to the standard of care. These trials, along with others such as TB PRACTECAL [11] have helped establish the safety and efficacy profiles of the BPaL regimen for DR-TB treatment, resulting in recent WHO recommendations for the programmatic use of this novel 6-month regimen [12].

Following clinical trials, several operational research programs have been and continue to be conducted to inform the programmatic implementation of BPaL. This work has focused on the optimization of patient selection criteria and the monitoring and management of potential side effects, but has not included evaluations assessing the cost-saving and cost-effectiveness potential of BPaL [13]. However, to date, two modelling studies suggest that BPaL-based regimens are likely to be both cost-saving and more effective than the current standard of care in high DR-TB burden settings [14–16].

Despite promising efficacy and economic results from clinical trials, operational research and modelling exercises, real-world cost-related evidence for BPaL is limited [14]. We aimed to estimate the patient and provider cost associated with BPaL compared to the current standard of care for the treatment of DR-TB in South Africa.

## Methods

### Design

Established in 2020, the South African BPaL clinical access program (CAP) (funded by the United States Agency for International Development (USAID)) is an open-label, single-arm intervention study with the goal to enrol up to 400 participants aged ≥14 years old who meet specific medical and clinical requirements including; a diagnosis of selected RR-TB, confirmed MDR-TB that is intolerant or unresponsive to conventional treatment regimens, or with RR/MDR-TB which is also resistant to any fluoroquinolone (pre-XDR) **(S1 Fig in S1 File)**. We conducted a socioeconomic sub-study in coordination with the BPaL CAP to estimate patient and provider costs of BPaL and current standard of care treatment regimens. Specifically, to estimate A) patient and B) provider costs, we conducted a cross-sectional patient cost survey and retrospective bottom-up and top-down costing analysis respectively.

### Setting, sites, and study participants

In evaluating both patient and provider costs, patients were enrolled at two participating sites within the South African BPaL CAP. Specifically, this included two government public hospitals with outpatient, decentralized DR-TB treatment facilities. These sites were located in high DR-TB burden districts and based on eligibility, provided care through the BPaL CAP or South African National TB Program.

### Standard of care treatment regimens

In South Africa, patients with RR/MDR-TB currently receive one of two standard of care treatment options: 1) a 9-11-month standard short oral regimen (SSOR) or 2) among patients with additional fluoroquinolone resistance, an 18-21-month standard long oral regimen (SLOR) or an individualised long regimen [17].

The SSOR includes an intensive phase comprising of bedaquiline used for 6 months; 400 mg once daily for two weeks followed by 200 mg three times a week for 22 weeks, in combination with linezolid for 2 months, and levofloxacin/moxifloxacin, ethambutol, high-dose isoniazid, pyrazinamide, and clofazimine used for 4 months with the possibility of extending to 6 months if the patient remains sputum smear positive at the end of 4 months. This is followed by a continuation phase, which includes levofloxacin/moxifloxacin, clofazimine, ethambutol and pyrazinamide for 5 months [17] **(Table 1).**

**Table 1:** Summary of treatment groups and regimens.

	Group 1: BPaL	Group 2: SSOR 9–11 months	Group 3: SLOR 18–21 months
**Indication**	FQ resistant FQ sensitive but treatment-intolerant or non-responsive	FQ sensitive	FQ resistance
**Regimen–early or intensive phase**	BDQ 400mg once daily for 2 weeks then 200mg 3 times per week plus PA 200mg once daily plus LZD 600mg once daily	4–6 months: LZD (2 months only)–BDQ (total 6 months)†–hdINH–LFX/M–CFZ–PZA–EMB^†^(4–6 months)	6–8 months: LZD–BDQ–LFX–DLM–TRD^†^
**Regimen–late or continuation phase**	BDQ 200mg 3 times per week plus PA 200mg once daily plus LZD 600mg once daily	5 months: LFX/M–CFZ–PZA–EMB	12 months: CFZ–TRD–LZD–BDQ and/or DLM
**Estimated treatment duration based on regimen**	24 weeks / 6 months	36–44 weeks / 9–11 months	72–84 weeks / 18–21 months

BPaL = bedaquiline, pretomanid & linezolid; SSOR = short all-oral regimen; SLOR = long all-oral regimen; FQ = fluoroquinolone; BDQ = bedaquiline; PA = Pretomanid; LZD = linezolid; hdINH = high-dose isoniazid; LFX = levofloxacin; M = moxifloxacin; CFZ = clofazimine; PZA = pyrazinamide; EMB = ethambutol; DLM = delamanid; TRD = terizidone; CAP = clinical access programme; TB = tuberculosis; MDR = multidrug-resistant; XDR = extensively drug-resistant.

^†^mid-point used as the duration for the phase.

The SLOR includes an intensive phase comprising of bedaquiline, linezolid, clofazimine, terizidone, and delamanid for 6 months, followed by a continuation phase of 12 months with three to four drugs including clofazimine, terizidone and one or two additional agents (linezolid, bedaquiline and/or delamanid, depending on tolerance). Patients with documented resistance to fluoroquinolones and bedaquiline and/or linezolid receive an individualized regimen based on the drug resistance pattern, prior drug exposure and toxicity [17] **(Table 1).**

### Treatment groups

We enrolled patients in the following treatment groups: Group 1) included patients who received BPaL as part of the CAP, Group 2) included patients who received the 9–11 month SSOR and Group 3) included patients who received the 18–21 month SLOR or individualized long regimen according to the South African national RR-TB treatment guidelines [17] (**Table 1**).

### A) Patient costs: Cross-sectional patient cost survey

To estimate patient costs, we conducted a cross-sectional patient cost survey. Eligible patients included those ≥14 years old with confirmed sputum pulmonary RR/MDR-TB, pre-XDR or XDR-TB who initiated treatment and were still receiving care at one of the participating study sites between 03/2021 and 11/2022, and who were willing to participate and provided written informed consent. Patient recruitment took place from 19/09/2022 to 20/12/2022.

#### Data collection

Based on the criteria detailed above, clinical teams at the study sites identified potentially eligible patients and referred them to the study team who confirmed eligibility and obtained written informed consent. As the BPaL regimen (Group 1) does not have a defined intensive or continuation phase like the SSOR and the SLOR (outlined in **Table 1**), for the administration of the patient cost survey, we defined an early (≥2 weeks but <4 months) and late phase (≥4 months) of treatment with BPaL. We used the average treatment duration outlined in **Table 1** for each phase within respective treatment groups.

Following the approach outlined in the WHO TB Patient Cost Handbook [18], at the time of the interview, patients were required to have been in their current treatment phase for a minimum of 14 days and were subsequently interviewed once only, either during the early/intensive (BPaL/SSOR or SLOR) or the late/continuation phase of treatment (BPaL/SSOR or SLOR). Briefly, patients report costs from the start of the current phase through to the interview date. These costs are then extrapolated for the remainder of the treatment phase so the total cost for the phase can be calculated [19]. This extrapolation assumes that all patients successfully complete the phase of treatment they were in when interviewed. While costs for respective patients in the non-surveyed phase are not collected during the interview, data from other patients are included to estimate the total cost of treatment across both phases and all treatment groups.

Patient costs were estimated according to WHO TB costing guidelines [18]. Direct (medical and non-medical) and indirect cost (lost income/opportunity costs) data were collected using the WHO generic patient cost instrument [18]. Demographic characteristics such as age, gender, employment prior to TB diagnosis and at the time of the interview, as well as TB history were collected. Surveys were administered by trained members of the study team who were fluent in local languages (e.g., isiZulu, Xhosa, Sotho, etc) and provided verbal translation of the English-based survey. Study data were collected and managed using REDCap (Research Electronic Data Capture), a secure, web-based platform and electronic data capturing tool hosted at the University of the Witwatersrand [20,21].

#### Data analysis

*Direct medical and non-medical costs*. Total direct costs were summed and disaggregated by costing category and treatment group.

Direct costs were categorized as medical and non-medical. Medical costs included consultations, laboratory tests, imaging/radiography, medications and other medical procedures, net of any reimbursements. Non-medical costs included transportation, food, nutritional supplementation and accommodation associated with seeking TB care [18]. These costing categories were used for TB-related follow-up visits (clinical and medication collection visits), hospital admissions and/or directly observed therapy (DOT) visits in both the early/intensive and late/continuation phases of treatment. Pre-treatment costs were not included in this analysis.

*Indirect costs*. Indirect costs included productivity and economic costs of TB patients as a result of TB healthcare visits and/or hospitalization during their TB episode [18]. Income loss was used as the primary measure of indirect costs for participants [22] and was calculated for the total time participants spent seeking care and treatment for TB (i.e., income earning hours lost). This was limited to patients employed prior to TB diagnosis and included travel, waiting, consultation, and hospitalized time.

Specifically, to calculate total patient time lost due to TB, the mean number of treatment visits per patient was multiplied by the mean visit duration within respective treatment phases. Thereafter, according to the human capital approach, total indirect patient costs were calculated as the product of total time lost due to TB and hourly rate [18] (monthly individual income prior to TB diagnosis divided by total monthly working hours). An adjusted human capital approach was applied to all patients using reported personal income/hourly wage for those employed prior to TB diagnosis and South African national minimum hourly wage (South African Rand (ZAR) 23.19 per hour) [23] for those unemployed prior TB diagnosis/missing personal income.

*Catastrophic costs*. Catastrophic costs were defined as the quotient of total treatment costs (direct and indirect costs) and annual household income prior to TB diagnosis exceeding various thresholds (15%, 20% and 25%) [18]:

$$Catastrophic\;costs=\frac{\;Total\;treatment\;costs\;(direct+indirect\;costs)}{Annual\;household\;income\;prior\;to\;TB\;diagnosis\;}>Threshold\;\%$$

Catastrophic costs were limited to patients who reported annual household income prior to TB diagnosis.

*Coping strategies*. Financial coping strategies utilized to finance the cost of patients’ TB treatment included the taking of interest-bearing loans, borrowing money from relatives and/or friends and/or selling property or assets (defined as dissaving) [18].

*Cost presentation*, *inflation and conversion*. Due to the generally skewed nature of patient cost data with most reported values being zero or close to zero, we summarized continuous cost estimates through arithmetic means. This approach is widely regarded as a robust methodological choice in health economics analyses and is favoured over the presentation of medians which may be limited in its descriptive usefulness [24–27].

All costs including direct and indirect costs, individual and household income and costs of coping strategies were originally reported and collected in South African ZAR. Costs reported prior to 2022 were first inflated to 2022, using the 2022 World Bank Gross Domestic Product (GDP) deflator, and then converted to United States Dollar (US$) using the average exchange rate in 2022 (US$ 1 = ZAR 16.37) (currency converter http://www.oanda.com/currency/converter). Analyses were performed using SAS 9.4 (SAS Institute Inc., Cary, NC, USA) and MS Excel (Microsoft, Redmond, WA, USA).

### B) Provider costs: Bottom-up and top-down costing analysis

To evaluate provider costs of BPaL and standard of care treatment regimens, we conducted a bottom-up and top-down costing analysis [28]. This included a retrospective medical record review at the two participating BPaL CAP sites described above through which patient-level resource-use was aggregated (bottom-up), as well as a facility-level financial record review at a single centralized government public hospital through which fixed and/or shared costs were ascertained and apportioned (top-down).

#### Data collection—medical record review

Medical records included in the retrospective record review were for patients ≥14 years with confirmed sputum pulmonary RR/MDR-TB, pre-XDR, or XDR-TB, who initiated treatment between 02/2021 and 12/2022 in one of the three treatment groups at either of the two participating BPaL CAP sites who had a treatment outcome assigned/sufficient follow-up for an outcome to be assigned. Medical records were reviewed consecutively from 12/2022 going backwards, which provided sufficient follow-up for all groups with the majority of visit data collected during 2022. We excluded patients who transferred facilities prior to reaching the study endpoint. Medical records were accessed from 19/09/2022 to 22/12/2022 and authors had access to information that could identify individual participants during and after data collection.

#### Data analysis—patient resource-use and variable costs

We estimated patient resource-use from the day of treatment initiation until a final outcome was assigned. In addition to demographics, clinical characteristics and treatment outcomes, we collected the number and type of visits (initiation, scheduled, unscheduled etc), type and frequency of healthcare providers seen (doctor, nurse, social worker etc), frequency of TB and non-TB laboratory tests performed (diagnostic and monitoring), total number of TB and ancillary/non-TB drugs dispensed, and total admission/inpatient days reported. Total variable costs were estimated as resource-use (total units/quantity) multiplied by the unit cost of the resource.

Specifically, staff time for visits was estimated using time and motion data, where study staff prospectively collected the start and end times of TB interactions with various client-facing healthcare providers. Staff costs per minute were calculated using public sector salaries reported for healthcare workers in 2021 (average across grades/notches per provider type) [29].

Drug costs were obtained from the publicly available National Department of Health’s master procurement catalogue [30]. Since pretomanid was not available through tender in South Africa at the time of the analysis (as it was donated to South Africa’s National TB Program), its unit price was obtained through personal communication with TB Alliance in June 2023. Laboratory test costs were obtained from the National Health Laboratory Service’s state price list for 2018 [31].

#### Data collection and analysis—fixed/shared costs

While resource usage was obtained from participating study sites, supplies, equipment and overhead costs as well as headcount data used to apportion these costs were collected from a single centralized government public TB hospital and specialized referral center located in Johannesburg, South Africa. We assumed these fixed/shared costs to be similar across government public hospitals.

Specifically, equipment was discounted based on useful life years which varied from 3–6 years depending on equipment type (electronic office equipment, furniture etc) [32]. Overheads included electricity, water, effluent, maintenance and security costs etc. All fixed/shared costs were apportioned by total facility headcount using total outpatient visits and inpatient days in 2022.

The cost of diagnosis for RR/MDR-TB, tracing activities, including follow-up calls or home visits for patients who were lost to follow-up (LTFU) and antiretroviral therapy (ART) for human immunodeficiency virus (HIV) co-infected patients, were not included in this analysis.

*Cost inflation and conversion*. All provider cost inflation and conversions followed the patient cost processes outlined above.

#### Treatment outcomes and cost-effectiveness

Treatment outcomes were consistent with standard case definitions and included favourable treatment outcomes (cure and treatment completion), and unfavourable treatment outcomes (death, LTFU, and treatment failure at 80 weeks) [33–35]. Using these outcomes, we estimated 1) mean “production costs” (the cost of producing a favorable outcome as defined above) and 2) incremental cost-effectiveness ratios (ICERs) defined as:

$$\begin{array}{l} ICER=\frac{\;\text{(}Cost\;A-Cost\;B\text{)}}{\text{(}Effectivness\;A-Effectivness\;B\text{)}} \end{array}$$

We consider ICERs against willingness to pay (WTP) thresholds set at 0.5 ($3,341), 1.0 ($6,681) and 1.5 ($10,022) times the GDP per capita [36] in South Africa in 2022 [37].

#### Statistical analysis and reporting—patient and provider costs

Study populations were described using descriptive statistics with mean and/or median measures of central tendency (standard deviations or 95% confidence intervals), frequencies and percentages, as appropriate. Direct and indirect costs for patients or guardians/carers were presented as means with standard deviations. Patients in the different treatment groups were compared using the chi-square test for categorical data and the Kruskal-Wallis test for continuous data.

#### Ethical consideration

This study was approved by the Human Research Ethics Committee of the University of the Witwatersrand (Protocol M220140). All patients provided written informed consent to participate in the patient cost survey. Although patients were recruited and interviewed during their routine clinic visits, visits took longer than normal, and participants were reimbursed ZAR150 (<$10) to cover out-of-pocket expenses and lost time while participating in the study. For the retrospective medical record review, there was no direct interaction with patients; however at treatment initiation, patients provided consent for their routine data to be evaluated.

## Results

### A. Patient costs

#### Cross-sectional patient cost survey—patient profile

Demographic and clinical characteristics of patients completing the patient cost survey are presented in **Table 2**. A total of 72 patients were interviewed and completed the patient cost survey. This included 30 (41.7%) patients in Group 1 (BPaL), 12 (16.7%) in Group 2 (SSOR) and 30 (41.7%) in Group 3 (SLOR). Mean age at the time of the interview was highest among patients on BPaL (48.0 years) and similar among those on the SSOR (35.3 years) and SLOR (34.8 years). One third of patients on BPaL were female (30.0%) compared to more than half on the SSOR (58.3%) and two thirds on the SLOR (66.7%). Levels of employment were relatively low among all patients, but highest among those on BPaL (26.7%) and lowest among those on the SLOR (17.9%). A majority of patients had an 8^th^ grade or higher level of education ranging from 80% among patients on BPaL to 100% among those on the SSOR and SLOR.

**Table 2:** Characteristics of patients included in the TB patient cost survey (N = 72).

		Treatment group (N, %)			
Characteristic	Description	BPaL	SSOR	SLOR	p-value
Regimen	Treatment group	30 (41.7%)	12 (16.7%)	30 (41.7%)	0.01^*^
Age, years	Mean (SD)	48.0 (11.5)	35.3 (8.4)	34.8 (7.5)	0.00^*⏊^
Gender	Female	9 (30.0%)	7 (58.3%)	20 (66.7%)	0.01^*^
Currently employed	Full or part-time^†^	8 (26.7%)	2 (18.2%)	5 (17.9%)	0.68
Education	More than Grade 8	24 (80.0%)	12 (100%)	30 (100%)	0.01^*^
Residence	Urban/sub-urban	7 (23.3%)	2 (16.7%)	5 (16.7%)	0.78
	Rural	23 (76.7%)	10 (83.3%)	25 (83.3%)	
Relationship	Single	15 (50.0%)	5 (45.5%)	15 (50.0%)	0.17
	Married/committed	11 (36.7%)	6 (54.6%)	15 (50.0%)	
	Divorced/separated	4 (13.3%)	0 (0.0%)	0 (0.0%)	
HIV status	Positive on ART	16 (53.3%)	8 (66.7%)	19 (63.3%)	0.61
	Negative	9 (30.0%)	4 (33.3%)	7 (23.3%)	
	Unknown/refuse	5 (16.7%)	0 (0.0%)	4 (13.3%)	
Patient category	New	17 (56.7%)	8 (66.7%)	15 (50.0%)	0.82
	Relapse	3 (10.0%)	1 (8.3%)	2 (6.7%)	
	Retreatment after LTFU/failure	10 (33.3%)	3 (25.0%)	13 (43.3%)	
Place of diagnosis	Primary health clinic	16 (55.2%)	8 (66.7%)	23 (76.7%)	0.22
	Hospital	13 (44.8%)	4 (33.3%)	7 (23.3%)	
Time to treatment initiation, days^‡^	Mean (SD)	12.9 (13.0)	5.7 (4.1)	19.5 (25.0)	0.08^⏊^
Drug-susceptibility	RR/MDR-TB	25 (86.2%)	11 (91.7%)	26 (86.7%)	0.88
	Pre-XDR/XDR	4 (13.8%)	1 (8.3%)	4 (13.3%)	
Interviewed in	Intensive/early	22 (73.3%)	6 (50.0%)	13 (43.3%)	0.06
	Continuation/late	8 (26.7%)	6 (50.0%)	17 (56.7%)	
Main income earner^±^	Patient	12 (92.3%)	2 (50.0%)	10 (66.7%)	0.14

TB = tuberculosis; BPaL = bedaquiline, pretomanid & linezolid; SSOR = standard short all-oral regimen; SLOR = standard long all-oral regimen; SD = standard deviation; HIV = human immunodeficiency syndrome; ART = antiretroviral therapy; LTFU = loss to follow-up; RR = rifampicin-resistant; MDR = multidrug-resistant; XDR = extensively drug-resistant.

^*^p≤0.05 using the using the chi-square test unless otherwise indicated; ^⏊^Kruskal-Wallis non-parametric test for continuous data.

^†^excluding students

^‡^time to treatment initiation defined at the interval between diagnosis and the start of TB treatment.

^±^prior to patients TB diagnosis.

At the time of the interview, 53.3%, 66.7% and 63.3% of patients on BPaL, the SSOR and SLOR were HIV-positive and on ART. More than half of all patients were diagnosed in a primary health facility ranging from 55.2% (BPaL) to 76.7% (SLOR) while mean time to treatment initiation ranged from 5.7 days (SSOR) to 19.5 days (SLOR). Most patients were classified with a RR/MDR-TB resistance pattern (86.2% to 91.7% for those on BPaL and the SSOR respectively). The majority of patients on BPaL were interviewed in the intensive/early phase of treatment (73.3%) while 50% or more of those on the SSOR (50.0%) and SLOR (56.7%) were interviewed in the continuation/late phase of treatment. Total time on treatment at the time of the interview was lowest among patients on BPaL (79.8 days) and increased among those on the SSOR (121.6 days) and SLOR (220.8 days) **(S1 Table in S1 File)**.

#### Direct, indirect and total on-treatment costs

Total on-treatment patient costs are presented in **Table 3**. Total mean on-treatment patient costs were lowest among those on BPaL ($56.6; SD $88.4) and quadrupled among those on the SSOR ($228.1; SD $287.8) and SLOR ($224.7 SD $268.6). Specifically, among those on BPaL, the SSOR and SLOR, direct medical costs were negligible at $0.9, $0.3 and $0.5 respectively, while direct non-medical costs generally accounted for the largest proportion of total costs (i.e., mean direct non-medical costs for BPaL ($54.6) was lower than the SSOR ($227.8) and SLOR ($224.3).

**Table 3:** Direct and indirect on-treatment costs of patients accessing TB care (USD 2022) (N = 72).

		Treatment group		
		BPaL (n = 30)	SSOR (n = 12)	SLOR (n = 30)
Cost type	Cost category	Total cost	Total cost	Total cost
Direct medical costs, mean (SD)	Medication, consultation, radiography and laboratory tests	$0 ($0)	$0 ($0)	$0 ($0)
	Procedures	$0.1 ($0.7)	$0 ($0)	$0 ($0)
	Hospitalisation	$1.4 ($6)	$0.6 ($1.4)	$0 ($0)
	Guardian costs (medication)	$0 ($0)	$0 ($0)	$2.7 ($6.1)
**Total, mean (SD)**		$0.9 ($4.5)	$0.3 ($0.9)	$0.5 ($2.5)
Direct non-medical costs, mean (SD)	Travel	$28.5 ($76.8)	$144.6 ($150.3)	$181.1 ($246.7)
	Accommodation	$0 ($0)	$0 ($0)	$13.0 ($53.5)
	Food/supplementation	$19.7 ($57.2)	$61.6 ($173.9)	$31.7 ($76.5)
	Guardian costs (accommodation, food, transport etc)	$27.5 ($32.1)	$129.8 ($79.8)	$16.7 ($21.5)
**Total, mean (SD)**		$54.6 ($88.6)	$227.8 ($288.0)	$224.3 ($268.9)
Indirect costs, mean (SD)	Income loss (time)^†^	$29.2 (-)^‡^	-^±^	-^±^
	*Income loss (time)* ^***¶***^	*$61*.*1 ($155*.*0)*	*$174*.*1 ($216*.*0)*	*$172*.*6 ($182*.*6)*
	Social welfare payment	$24.2 ($73.8)	$252.0 ($311.4)	$306.4 ($540.7)
**Total, mean (SD)** ^**§**^		$1 ($5.3)	$0 ($0)	$0 ($0)
***Total*, *mean (SD)*** ^***#***^		*$43*.*4 ($135*.*1)*	*$69*.*7 ($83*.*5)*	*$81*.*4 ($149*.*4)*
**Total episode costs (direct medical + direct non-medical + indirect costs**				
**Total on-treatment costs, mean (SD)** ^*^		$56.6 ($88.4)	$228.1 ($287.8)	$224.7 ($268.6)
***Total on-treatment costs*, *mean (SD)*** ^**^		*$99*.*0 ($166*.*9)*	*$297*.*7 ($345*.*7)*	*$306*.*2 ($367*.*2)*

TB = tuberculosis; USD = United States Dollar; BPaL = bedaquiline, pretomanid & linezolid; SSOR = standard short all-oral regimen; SLOR = standard long all-oral regimen; SD = standard deviation.

^†^Only among patients employed prior to TB diagnosis using reported personal income/hourly wage prior to diagnosis (WHO human capital approach).

^‡^n = 1; ^±^n = 0.

^¶^Among all patients using reported personal income/hourly wage for those employed prior to TB diagnosis and South African national minimum hourly wage ($1.42) for those unemployed prior TB diagnosis/missing personal income (adjusted WHO human capital approach).

^§^Total indirect costs calculated as income loss (time) as per the WHOs human capital approach net of social welfare payments (negative values adjusted to $0).

^***#***^Total indirect costs calculated as income loss (time) as per the WHOs adjusted human capital approach net of social welfare payments (negative values adjusted to $0).

^*^Direct medical costs + direct non-medical costs + indirect costs (human capital approach).

^**^Direct medical costs + direct non-medical costs + indirect costs (adjusted human capital approach).

Direct non-medical costs were driven primarily by travel and food/nutritional supplementation costs, the means of which were lower among patients on BPaL ($28.5 and $19.7) compared to those on the SSOR ($144.6 and $61.6) and SLOR ($181.1 and $31.7).

Based on the human capital approach and as a result of high rates of pre-diagnosis unemployment/missing personal income, indirect costs were negligible across treatment groups. However, when adjusting for high rates of unemployment/missing personal income through the use of the South African national minimum hourly wage (adjusted human capital approach), indirect costs increased in all treatment groups but were lowest among those on BPaL ($61.1) and tripled among those on the SSOR ($174.1) and SLOR ($172.6). Social welfare payments were lowest among patients on BPaL ($24.2) and increased among those on the SSOR ($252.0) and SLOR ($306.4). Being a South African citizen, between 18–59 years old and meeting the requirements of the means test (i.e., income and assets of the applicant below a defined threshold), and not receiving another grant are some of the eligibility requirements for a Social Relief of Distress Grant (SRD). Shorter treatment duration and an earlier return to labor-related activities and/or less severe disability could possibly explain why fewer patients on BPaL were recipients of such social welfare.

#### Support and coping

Catastrophic and coping costs are presented in **Table 4**. Self-reported annual household income prior to TB diagnosis, though reported by less than half of all patients, was highest among those on BPaL ($8,452.6) and decreased among patients on the SSOR ($7,220.5) and SLOR ($6,326.7). No patients on BPaL or the SSOR experienced catastrophic costs across any threshold (15%, 20% or 25%) while 20.0% and 6.7% of patients on the SLOR experienced catastrophic costs at the 15% and 20% and 25% thresholds, respectively.

**Table 4:** Catastrophic and coping costs of patients accessing TB care (USD 2022) (n = 72).

		Treatment group			
Indicator	Description	BPaL (n = 30)	SSOR (n = 12)	SLOR (n = 30)	p-value
**Household income**	Annual household income prior to TB diagnosis, mean (SD)	$8,452.6 ($8,676.9)	$7,220.5 ($3,772.9)	$6,326.7 ($6,022.0)	0.73^⏊^
	Missing	17 (56.7%)	8 (66.7%)	15 (50.0%)	
**Catastrophic costs (% threshold)**	15%	0 (0%)	0 (0%)	3 (20%)	0.15
	20%	0 (0%)	0 (0%)	1 (6.7%)	0.56
	25%	0 (0%)	0 (0%)	1 (6.7%)	0.56
**Coping mechanisms**	Borrowed	8 (26.7%)	8 (66.7%)	17 (56.7%)	0.01^*^
	Sold assets	1 (3.6%)	0 (0%)	0 (0%)	0.48
	Dissaving^†^, mean (SD)	$35.8 ($27.8)	$42.1 ($65.5)	$152.9 ($454.0)	0.75^⏊^
**Perceived financial impact on the household**	No impact	6 (20%)	0 90%)	0 (0%)	0.03^*^
	Little to moderate	3 (10%)	1 (8.3%)	6 (20%)	
	Serious to very serious	21 (70%)	11 (91.7%)	24 (80%)	
**Social consequences**	Social exclusion	13 (43.3%)	6 (50%)	22 (73.3%)	0.06
	Food insecurity	9 (30%)	6 (50%)	19 (63.3%)	0.03^*^
	Job loss	7 (23.3%)	2 (16.7%)	7 (23.3%)	0.88
	Interrupted schooling^‡^	0 (0%)	2 (16.7%)	2 (6.7%)	0.10
	Divorce	1 (3.3%)	0 (0%)	1 (3.3%)	0.81
	Any of the above	18 (60%)	10 (83.3%)	25 (83.3%)	0.09

TB = tuberculosis; USD = United States Dollar; BPaL = bedaquiline, pretomanid & linezolid; SSOR = standard short all-oral regimen; SLOR = standard long all-oral regimen; SD = standard deviation; E = early/intensive phase; L = late/continuation phase.

^*^p≤0.05 using the using the chi-square test unless otherwise indicated; ^⏊^Kruskal-Wallis non-parametric test for continuous data.

^†^dissaving = total amount/s borrowed + total amount/s received from the sale of asset/s.

^‡^of children residing in the household.

Coping mechanisms including borrowing money to finance TB treatment was lowest among those on BPaL (26.7%), and more than doubled among those on the SSOR (66.7%) and SLOR (56.7%) (p≤0.05). Similarly, related dissaving’s (amounts borrowed + value of assets sold) was lowest among patients treated with BPaL ($35.8) and increased with treatment duration among patients on the SSOR ($42.1) and SLOR ($152.9).

More than two out of three patients on BPaL (70.0%), the SSOR (91.7%) and SLOR (80.0%) perceived their TB disease as having a serious/very serious financial impact on their household (p≤0.05). Additionally, the incidence of social consequences such as social exclusion and food insecurity was lowest among those on BPaL (43.3% and 30.0% respectively) and increased with the SSOR (both 50.0%) and SLOR (73.3% and 63.3% respectively) with the latter being statistically higher than BPaL (p≤0.05).

### B. Provider costs

#### Retrospective medical record review patient profile

Demographic and clinical characteristics of patients included in the retrospective medical record review are presented in **Table 5**. Resource-use and clinical outcome data were extracted from a total of 112 medical records. This included 42 (37.5%) patients in Group 1 (BPaL), 46 (41.1%) in Group 2 (SSOR) and 24 (21.4%) in Group 3 (SLOR). Patients on BPaL were older (43.9 years) compared to those on the SSOR (37.9 years) and SLOR (37.1 years). Fewer than half of all patients on BPaL, the SSOR and SLOR were female (11.9%, 37.0% and 25.0% respectively), while at least half were newly diagnosed (54.8%, 73.9% and 50.0% respectively). Among those with a favourable treatment outcome, days admitted while on treatment was similar between patients on BPaL and the SSOR (43.8 days and 43.7 days respectively), but higher for those on the SLOR (63.5 days). The number of outpatient visits was lowest among patients on BPaL (7.3 visits) and increased among those on the SSOR (8.9 visits) and SLOR (14.6 visits). Favourable treatment outcomes were highest among patients on BPaL (92.9%), followed by those on the SSOR (67.4%) and SLOR (41.7%).

**Table 5:** Characteristics of patients included in the retrospective medical record review to assess patient resource-use (n = 112).

Characteristic	Description	Treatment group			
Regimen	Treatment group	BPaL (n = 42)	SSOR (n = 46)	SLOR (n = 24)	p-value
**Age, years**	Mean (SD)	43.9 (12.0)	37.9 (11.5)	37.1 (10.6)	0.01^*⏊^
**Gender**	Female	5 (11.9%)	17 (37.0%)	6 (25.0%)	0.03^*^
**Patient category**	New	23 (54.8%)	34 (73.9%)	12 (50.0%)	0.12
	Relapse	12 (28.6%)	5 (10.9%)	5 (20.8%)	
	Retreatment after LTFU/failure	7 (16.7%)	7 (15.2%)	7 (29.2%)	
**Outcomes**					
**Clinical outcomes**	Died	2 (4.8%)	6 (13%)	5 (20.8%)	0.14
	LTFU	1 (2.4%)	8 (17.4%)	7 (29.2%)	0.01^*^
	Complete	15 (35.7%)	3 (6.5%)	3 (12.5%)	0.00^*^
	Cured	24 (57.1%)	28 (60.9%)	7 (29.2%)	0.03^*^
	Missing	0 (0%)	1 (2.2%)	2 (8.3%)	0.13
**Study outcomes**	Favorable^‡^	39 (92.9%)	31 (67.4%)	10 (41.7%)	0.00^*^
	Unfavorable	3 (7.1%)	15 (32.6%)	14 (58.3%)	
**Treatment visit type**					
**All patients** ^†^	Inpatient days, mean (SD)	41.0 (43.8)	41.7 (41.6)	65.6 (58.4)	0.21^⏊^
	Outpatient visits, mean (SD)	6.9 (3.4)	7.5 (4.7)	8.0 (7.2)	0.98^⏊^
**Favourable outcomes** ^‡^	Inpatient days, mean (SD)	43.8 (44.2)	43.7 (43.5)	63.5 (58.6)	0.61^⏊^
	Outpatient visits, mean (SD)	7.3 (3.1)	8.9 (4.8)	14.6 (5.1)	0.00^*⏊^

BPaL = bedaquiline, pretomanid & linezolid; SSOR = standard short all-oral regimen; SLOR = standard long all-oral regimen; SD = standard deviation; LTFU = loss to follow-up.

^*^p≤0.05 using the using the chi-square test unless otherwise indicated; ^⏊^Kruskal-Wallis non-parametric test for continuous data.

^†^irrespective of outcome.

^‡^completed/cured treatment only.

#### Cost and cost-effectiveness

Provider costs for TB treatment by outcome are presented in **Table 6**. Total cost of treatment provision was marginally higher for patients on BPaL ($4,643.2 per patient) compared to those on the SSOR ($4,563.8 per patient) with both regimens costing substantially less than treatment with the SLOR ($7,483.2 per patient). Resulting from increased resource-use in the CAP, the main cost drivers leading to a higher treatment cost among patients on BPaL compared to those on the SSOR included, client facing staff time ($382.8 vs. $296.7 respectively), TB drugs ($1,178.4 vs. $1,099.9 respectively) and laboratory tests ($44.2 vs. $35.5 respectively), which may all likely be lower under programmatic conditions.

**Table 6:** Provider costs for TB provision by clinical outcome (mean, calculated 95% CI) (USD 2022).

	All outcomes (n = 112)		
	Treatment group		
**Cost category**	**BPaL (n = 42)**	**SSOR (n = 46)**	**SLOR (n = 24)**
TB diagnostics/monitoring	$61.5 ($60.9-$62.1)	$66.7 ($66.1-$67.3)	$79.9 ($79.3-$80.5)
Client-facing providers	$382.8 ($382.2-$383.4)	$296.7 ($296.1-$297.3)	$341.6 ($341.0-$342.2)
Drugs (TB)	$1,178.4 ($1,177.8-$1,179.0)	$1,099.9 ($1,099.3-$1,100.5)	$2,254.1 ($2,253.5-$2,254.7)
*Drugs (TB) considering BDQ price reduction (sensitivity analysis)* ^†^	*$978*.*3 ($977*.*7-$978*.*9)*	*$846*.*3 ($845*.*7-$846*.*9)*	*$1*,*973*.*5 ($1*,*972*.*7-$1*,*974*.*3)*
Drugs (non-TB)	$3.8 ($3.2-$4.4)	$31.7 ($31.1-$32.3)	$32.5 ($31.9-$33.1)
Laboratory tests	$44.2 ($43.6-$44.8)	$35.5 ($34.9-$36.1)	$54.7 ($54.1-$55.3)
Fixed costs/overhead	$2,821.9 ($2,821.3–2,822.5)	$2,879.8 ($2,879.2-$2,880.4)	$4,480.8 ($4,480.2-$4,481.4)
Equipment	$150.7 ($150.1-$151.3)	$153.7 ($153.1-$154.3)	$239.7 ($239.1-$240.3)
**Total cost per patient treated, mean (95% CI)**	$4,643.2 ($4,642.6-$4,643.8)	$4,563.8 ($4,563.2-$4,564.4)	$7,483.2 ($7,482.6-$7,483.8)
***Total cost per patient treated*, *mean (95% CI)*** *(sensitivity analysis estimate)*^†^	*$4*,*443*.*1 ($4*,*442*.*5-$4*,*443*.*7)*	*$4*,*310*.*3 ($4*,*309*.*7-$4*,*310*.*9)*	*$7*,*202*.*7 ($7*,*201*.*9-$7*,*203*.*5)*
	**Favorable outcomes (n = 80)**		
	**Treatment group**		
**Cost category**	**BPaL (n = 39)**	**SSOR (n = 31)**	**SLOR (n = 10)**
TB diagnostics/monitoring	$65.6 ($65.0-$66.2)	$74.6 ($74.0-$75.2)	$150.7 ($150.1-$151.3)
Client-facing providers	$406.9 ($406.3-$407.5)	$305.8 ($305.2-$306.4)	$422.7 ($422.1-$423.3)
Drugs (TB)	$1,249.4 ($1,248.8-$1,250.0)	$1,256.5 ($1,255.9-$1,257.1)	$3,587.3 ($3,586.7-$3,587.9)
*Drugs (TB) considering BDQ price reduction (sensitivity analysis)* ^†^	*$1*,*039*.*4 ($1*,*038*.*8-$1*,*040*.*0)*	*$949*.*3 ($948*.*6-$950*.*0)*	*$3*,*105*.*8 ($3*,*104*.*4-$3*,*107*.*2)*
Drugs (non-TB)	$4.1 ($3.5-$4.7)	$36.9 ($36.3-$37.5)	$45.3 ($44.7-$45.9)
Laboratory tests	$46.3 ($45.7-$46.9)	$44.0 ($43.4-$44.6)	$60.1 ($59.5-$60.7)
Fixed costs/overhead	$3,015.4 ($3,014.8-$3,016)	$3,026.4 ($3,025.8-$3,027.0)	$4,418.7 ($4,418.1-$4,419.3)
Equipment	$161.0 ($160.4-$161.6)	$161.3 ($160.7-$161.9)	$235.3 ($234.7-$235.9)
**Total cost per favourable outcome, mean (95% CI)**	$4,948.7 ($4,948.1-$4,949.3)	$4,905.6 ($4,905.0-$4,906.2)	$8,919.9 ($8,919.3-$8,920.5)
***Total cost per favourable outcome*, *mean (95% CI)*** *(sensitivity analysis estimate)*^†^	*$4*,*738*.*7 ($4*,*738*.*1-$4*,*739*.*3)*	*$4*,*598*.*3 ($4*,*597*.*6-$4*,*599*.*0)*	*$8*,*438*.*5 ($8*,*437*.*1-$8*,*439*.*9)*

TB = tuberculosis; USD = United States Dollar; CI = confidence interval; BPaL = bedaquiline, pretomanid & linezolid; SSOR = standard short all-oral regimen; SLOR = standard long all-oral regimen; BDQ = bedaquiline.

^†^unit price of bedaquiline 100mg tablet changed from $1.02 (USD 2022) at the time of treatment to $0.65 (USD 2024) per unit (tablet) based on Global Drug Facility price reduction (2023).

When limited to patients with favorable treatment outcomes, production costs (cost per favorable outcome) were similar among those on BPaL ($4,948.7 per patient) and SSOR ($4,905.6 per patient) with costs increasing substantially among those on the SLOR ($8,919.9 per patient).

Cost-effectiveness estimates for BPaL, the SSOR and SLOR regimens are presented in **Table 7**. Treatment with the SLOR proved the costliest and least effective regimen and as such, was dominated by shorter treatment alternatives (eliminated from consideration) [38]. At all recognized WTP thresholds of 0.5 ($3,341), 1.0 ($6,681) and 1.5 ($10,022) times GDP per capita in South Africa (2022), treatment with BPaL (intervention) was more cost-effective than treatment with the SSOR comparator (ICER: $311.4 < WTP threshold specified) [38]. In an additional sensitivity analysis accounting for the Global Drug Facilities recent price reduction of bedaquiline (unit price of bedaquiline 100mg tablet changed from $1.02 (USD 2022) at the time of treatment to $0.65 (USD 2024) per unit (tablet) based on Global Drug Facility price reduction (2023), treatment with BPaL still proved more cost-effective than treatment with the SSOR (ICER: $520.8 < WTP threshold specified) while total treatment costs decreased across all treatment groups.

**Table 7:** Cost-effectiveness of the BPaL treatment regimen (USD 2022).

Treatment group	Effectiveness (favourable outcome/total)	Total cost per patient treated (mean)	ICER	WTP (GDP per capita USD 2022)
**BPaL (intervention)**	0.929 (39/42)	$4,643.2	$311.4	$6,681^⏊^; $3,341^‡^; $10,022^±^
**SSOR (comparator)**	0.674 (31/46)	$4,563.8	-	-
**SLOR (comparator)**	0.417 (10/24)	$7,483.2	Dominated^†^	-
***Sensitivity analysis (considering 2023 Global Drug Facility bedaquiline price reduction)*** ^¶^				
**Treatment group**	**Effectiveness (favourable outcome/total)**	**Total cost per patient treated (mean)**	**ICER**	**WTP (GDP per capita USD 2022)**
**BPaL (intervention)**	0.929 (39/42)	$4,443.1	$520.8	$6,681^⏊^; $3,341^‡^; $10,022^±^
**SSOR (comparator)**	0.674 (31/46)	$4,310.3		
**SLOR (comparator)**	0.417 (10/24)	$7,202.7	Dominated^†^	

BPaL = bedaquiline, pretomanid & linezolid; USD = United States Dollar; ICER = incremental cost-effectiveness ratio; WTP = willingness to pay; GDP = gross domestic product; SSOR = standard short all-oral regimen; SLOR = standard long all-oral regimen.

^†^eliminate from consideration due to lowest effectiveness and highest cost.

^‡^0.5 times GDP per capita (low threshold).

^±^1.5 times GPD per capita (high threshold).

^⏊^1.0 times GDP per capita.

^¶^unit price of bedaquiline 100mg tablet changed from $1.02 (USD 2022) at the time of treatment to $0.65 (USD 2024) per unit (tablet) based on Global Drug Facility price reduction (2023).

## Discussion

We conducted a cross-sectional patient cost survey and bottom-up and top-down costing analysis to estimate the costs of DR-TB treatment in South Africa’s public sector. From the patient perspective, treatment with the new 6-month BPaL regimen costs approximately 75% less than the SSOR and SLOR respectively. Direct medical costs were negligible across all three treatment regimens, which is consistent with free public healthcare in the country, while direct non-medical costs accounted for the largest proportion of total treatment costs. Patients on BPaL had fewer follow-up treatment visits and consequently incurred substantially lower non-medical costs like travel and food/nutritional supplementation costs.

We calculated indirect costs using the human capital approach (i.e., time used while seeking and receiving care during the TB episode [in hours] multiplied by an individual hourly income). Because self-reported household income was poorly reported (>55% missing), we opted not to use the output approach as an alternative to measuring indirect costs. Additionally, while patients on BPaL had fewer outpatient visits and income hours lost (16.9 visits and 32.6 hours) compared to those on the SSOR (28.3 visits and 107.5 hours) and SLOR (34.1 visits and 85.0 hours) **(S1 Table in S1 File)**, due to high levels of pre-diagnosis unemployment, resulting indirect costs were negligible, but may be higher in other samples.

When considering patient costs over time and to address the limitations of a cross-sectional patient cost survey (one interview per patient per phase with costs extrapolated for the phase and episode) we constructed an early and late treatment phase for those on BPaL. While higher costs in the continuation phase may be due to the accumulation of costs over a longer period, monthly costs are typically lower in the continuation phase compared with the intensive phase [39]. It should be noted that more patients in the SLOR (57% versus 27% in BPaL and 50% in SSOR) were interviewed later (~26 weeks) into the continuation phase of treatment. This, according to Bengey and colleagues, may not be optimal as interviewing patients during the initial stage of the continuation phase provides more accurate estimates [19]. Therefore, because we interviewed SLOR patients later in the continuation phase due to the inherently prolonged treatment duration, this may have underestimated the total cost of the phase, the total cost of treatment as well as the perceived financial impact. Similarly, because we interviewed more BPaL patients in the intensive phase, total costs on this regimen may have been overestimated [19]. Considering this caveat, the proportion of patients who experienced catastrophic costs or used dissaving and the monetary value thereof, appear to be essential measures of financial impact.

No patients on BPaL or the SSOR experienced catastrophic costs at any threshold while this increased to one in five patients on the SLOR at a 15% threshold. While annual household income prior to TB diagnosis was higher among patients on BPaL and the SSOR compared to the SLOR, these differences were not statistically significant and proportions of catastrophic costs may be attributable primarily to variations in treatment costs rather than variations in income.

Given the difficulties in collecting reliable income data [40] used in measuring catastrophic costs we also consider coping mechanisms such as borrowing money and selling assets (dissaving) as proxy indicators of financial hardship [41]. Patients on BPaL not only utilized these coping mechanisms less frequently than those on the SSOR and SLOR, but did so at a lower total monetary value.

While reported rates of serious/very serious financial impact due to their TB disease was relatively high for all patients in this study, this rate was lower for those on BPaL than those on the SSOR and SLOR. Using informative indicators such as catastrophic costs, coping strategies and perceived financial impact of TB, patients on BPaL were less affected financially by the disease than those on the SSOR or SLOR.

From the provider perspective, 93% of patients on BPaL had a favourable outcome, consistent with the results from the NixTB [2] (90%) and ZeNIX [10] (93%) clinical trials. In contrast, those on the SSOR and SLOR achieved lower favorable outcome rates (67% and 42%, respectively), which are comparable to the success rates reported in long-term cohort studies from South Africa (69% and 58%, respectively) [42]. While total provider costs for treatment provision through BPaL and the SSOR was similar, we found that BPaL was more cost-effective than both current standard of care options (SSOR and SLOR).

Costs are influenced by a number of factors including; the model of care (inpatient > outpatient), setting (rural > urban), HIV co-infection (HIV/TB > TB alone) and the treatment regimen (long > short) [22,43,44]. It is important to note that in this study, BPaL was implemented under ‘research conditions’ of the BPaL CAP, while treatment with the SSOR and SLOR were administered under routine programmatic conditions. Different selection criteria (i.e., intolerant or unresponsive to conventional MDR-TB treatment), more specialized personnel, and additional resources resulted in extra treatment costs of BPaL as provided through the CAP. Between treatment provision with BPaL and the SSOR, we found that staff time accounted for the largest difference in cost (8.2% and 6.5% of total treatment cost respectively). This additional staff or client-facing time is likely due to the safety and reporting requirements of the CAP, and will likely decrease in frequency when BPaL is administered in routine care. Thereafter, with sustained improvement in treatment outcomes under routine programmatic conditions, the cost-effectiveness of BPaL compared to the SSOR and SLOR is likely to increase further.

Similarly, on further examination, BPaL CAP protocols require hospitalizing patients for two weeks wherever feasible at the start of treatment so that drugs can be administered under supervision [45] rather than hospitalizing patients because it is required (i.e., adverse events, etc). This adds to the total cost observed for patients and the health system, and are therefore likely to be lower under programmatic condition where these additional inpatient days (mainly during early treatment) are not required.

### Limitations

In estimating both patient and provider costs we include patients from only two participating sites within the BPaL CAP. However, while patient experience and cost may differ based on setting, treatment provision as prescribed by the National Department of Health is likely to remain fairly consistent across the country. Subsequently, while patient costs in this study may not be representative of the collective DR-TB patient experience, provider cost estimates may be a more accurate reflection of national health expenditure.

With regard to patient costs, compared to longitudinal surveys, the well-known limitations of cross-sectional surveys [18] apply to our results and include; recall bias leading to inaccurate accounts of direct and indirect treatment costs, the use of a single survey response in only one of the two potential treatment phases, and the ‘crude extrapolation’ of costs across a given treatment phase. Consequently, inaccurate accounts of costs, including both under- and over-estimation may be magnified through extrapolation, and variation and/or nuances within a given treatment phase may not have been accounted for. Other limitations of the cross-sectional survey include sampling only individuals diagnosed with TB within the National Treatment Program network and not including any costs incurred beyond treatment completion.

To minimize the limitations associated with cross-sectional surveys, we followed the methodology prescribed in WHO TB Patient Cost Handbook [18] and attempted to interview an equal number of patients across treatment groups and treatment phases (early/intensive vs. late/continuation) to obtain more accurate cost estimates within respective phases and subsequently more accurate estimates of total treatment costs.

While longitudinal surveys may result in more accurate cost estimates, they are considerably more expensive, labour-intensive and burdensome to administer [19] and coupled with the prolonged duration of standard of care regimens (up to 21 months) proved impractical in this study.

Due to the relatively small sample size informing the patient cost analysis, associated cost estimates are subject to a large or high standard deviation indicating a high degree of variance around the mean in the observed data. As such, these results should be interpreted with caution.

It should be noted that the BPaL CAP had more stringent eligibility criteria than the routine program, so while we compared characteristics at treatment initiation, the groups may differ by other characteristics (e.g., pregnant women, substance abuse, depression), which may have influenced treatment outcomes. Fewer than half (33.3%-50.0%) reported individual or household income, limiting the ability to calculate catastrophic costs and alternative approaches for lost income (i.e., the output approach). Proxy measures were included and discussed where appropriate, to address this limitation.

With regard to provider costs, while we account for all DR-TB medications administered to patients while on treatment, we did not directly include the cost of managing adverse reactions commonly associated with second-line therapies [7]. However, increased resource-use by way of more frequent monitoring through healthcare interactions, laboratory tests, and drug-substitutions/additions would have been reflected in the medical record review and subsequently accounted for in the final provider cost estimates.

## Conclusion

Resulting primarily from reductions in both direct non-medical costs such as transportation, food and nutritional supplementation as well as indirect costs such as income hours lost while seeking TB care, total patient costs on the newly recommended 6-month BPaL regimen decreased by 75% compared to current standard of care treatment options in South Africa. Additionally, while the cost of treatment provision through BPaL and the countries next best option—a 9-11-month regimen are similar, treatment with BPaL proves to be a more cost-effective option and will likely continue to be so under routine care. The above costs and cost-effectiveness estimates will be used in a budget impact analysis to model the costs and outcomes of BPaL/L (levofloxacin instead of moxifloxacin), according to the new South African MDR-TB treatment guidelines [36].

## Supporting information

S1 Checklist CHEERS checklist. (DOCX)

S1 File Supplementary tables and figures. (DOCX)
