# Supplementary material for: Patient and provider costs of the new BPaL regimen for drug-resistant tuberculosis treatment in South Africa: A cost-effectiveness analysis
Source: PLoS One. 2024 Oct 21;19(10):e0309034. doi: 10.1371/journal.pone.0309034 (PMC11493257; doi:10.1371/journal.pone.0309034)
Supplement: S1 File — (DOCX) [file pone.0309034.s002.docx]

**Supplementary Fig 1. South African BPaL Clinical Access Programme inclusion/exclusion criteria**

| **Inclusion criteria** |
| --- |
| 1. Provide written, informed consent prior to all study-related procedures (if under 18, include consent of legal guardian). 2. Body weight of ≥35 kg (in light clothing and no shoes). 3. Willingness and ability to attend scheduled follow-up visits and undergo study assessments. 4. Provide consent to HIV testing (if an HIV test was performed within 1 month prior to study start, it should not be repeated as long as documentation can be provided [ELISA and/or Western Blot]. If HIV status is a confirmed known positive, repeated HIV test is not needed provided documentation is available.) 5. Male or female, aged 14 years or above. 6. Participants with one of the following pulmonary TB conditions: 7. Pre-XDR TB with documented resistance to rifamycin and a fluoroquinolone at any time or at screening by any approved method- genotypic or phenotypic; 8. RR-TB with documented resistance to rifamycin in selected patients who the investigator thinks will benefit from a shorter all-oral regimen. 9. Chest X-ray picture (taken within three months prior to screening) consistent with pulmonary TB in the opinion of the Investigator. 10. Be of non-childbearing potential or using effective methods of birth control, as defined below:   **Non-childbearing potential:**   1. Participant - not heterosexually active or practices sexual abstinence; or 2. Female participant/sexual partner - bilateral oophorectomy, bilateral tubal ligation, and/or hysterectomy or has been postmenopausal with a history of no menses for at least 12 consecutive months; or 3. Male participant/sexual partner - vasectomized or has had a bilateral orchiectomy minimally three months prior to Screening.   **Effective birth control methods:**  A double contraceptive method should be used as follows:   1. Double barrier method which can include any two of the following: a male condom, diaphragm, cervical cap, or female condom (male and female condoms should not be used together); or 2. Barrier method (one of the above) combined with hormone-based contraceptives (with close monitoring for hepatotoxicity) or an intra-uterine device for the female participant/partner; 3. And are willing to continue practicing birth control methods throughout treatment and for 6 months (both male and female participants) after the last dose of study medication or discontinuation from study medication in case of premature discontinuation. |
| **Exclusion criteria** |
| **Medical History**   1. History of allergy or known hypersensitivity to any of the Investigational Medicinal Products or related substances. 2. Having participated in other clinical studies with dosing of investigational agents within 8 weeks prior to study start or currently enrolled in an investigational study that includes treatment with medicinal agents. Participants who are participating in observational studies or who are in a follow-up period of a study that included drug therapy may be considered for inclusion. 3. Significant cardiac arrhythmia requiring medication. 4. Participants with the following at Screening:    1. QTcF interval on ECG >500 msec    2. History of additional risk factors for Torsade de Pointes, (e.g., heart failure, hypokalaemia, family history of Long QT Syndrome);    3. Clinically significant ventricular arrhythmias; 5. Females who have a positive pregnancy test at Screening or are already known to be pregnant, breast-feeding, or planning to conceive a child during the study or within 6 months of cessation of treatment.   Males planning to conceive a child during the study or within 6 months of cessation of treatment.   1. A peripheral neuropathy of Grade 3 or 4, according to DMID (Appendix 2). Or, participants with a Grade 1 or 2 neuropathy that is likely to progress/worsen over the course of the study, in the opinion of the Investigator.   **Specific Treatments**   1. Concomitant use of Monoamine Oxidase Inhibitors (MAOIs) or prior use within 2 weeks of treatment assignment. 2. Concomitant use of serotonergic antidepressants or prior use within 3 days of treatment assignment if Investigator foresees potential risks for serotonin syndrome when combined with linezolid. 3. Concomitant use of any drug known to prolong QTc interval (including, but not limited to,- amiodarone, bepridil, chloroquine, chlorpromazine, cisapride, cyclobenzaprine, clarithromycin, disopyramide, dofetilide, domperidone, droperidol, erythromycin, fluoroquinolones, halofantrine, haloperidol, ibutilide, levomethadyl, mesoridazine, methadone, pentamidine, pimozide, procainamide, quinidine, sotalol, sparfloxacin, thioridazine). 4. Concomitant use of any drug known to induce myelosuppression. 5. Use of any drugs or substances within 30 days prior to dosing known to be strong inhibitors or inducers of cytochrome P450 enzymes (including but not limited to quinidine, tyramine, ketoconazole, fluconazole, testosterone, quinine, gestodene, metyrapone, phenelzine, doxorubicin, troleandomycin, cyclobenzaprine, erythromycin, cocaine, furafylline, cimetidine, dextromethorphan).   Exceptions may be made for participants that have received 3 days or less of one of these drugs or   1. Participants may have previously been treated for DS/MDR-TB (with specific exceptions for bedaquiline and/or linezolid as noted below) 2. Participants should not receive more than 4 weeks of bedaquiline or linezolid prior to enrolment/first dose of BPaL.   **Based on Laboratory Abnormalities**   1. Participants with the following toxicities at Screening (labs may be repeated) as defined by the enhanced Division of Microbiology and Infectious Disease (DMID) adult toxicity table (November 2007):    1. Haemoglobin level < 8.0 g/dl    2. Platelets grade 2 or greater (<75,000/mm3);    3. Absolute neutrophil count (ANC) <1000/ mm3    4. Alanine aminotransferase - Grade 3 or greater (> 3.0 x ULN) to be excluded    5. Serum creatinine level greater than 2 times upper limit of normal |

**Supplementary Table 1. Summary of self-reported visit information by treatment group used in the patient cost analysis (N=72).**

| **Characteristic** | **Variable** | **BPaL (n=30)** | **SSOR (n=12)** | **SLOR (n=30)** | **p-value** |
| --- | --- | --- | --- | --- | --- |
| **Time in treatment phase** | Time on treatment at interview:  Days, mean (SD) | 79.8 (60.2) | 121.6 (90.6) | 220.8 (184.6) | 0.01* |
| **Hospitalization** | Hospitalized at time of interview, N (%) | 8 (26.7%) | 1 (8.3%) | 10 (33.3%) | 0.25 |
|  | Previously hospitalized during current phase, N (%) | 12 (40.0%) | 4 (33.3%) | 14 (46.7%) | 0.71 |
|  | Mean duration of hospitalization during current phase (previous and current): Days, mean (SD) | 39.1 (21.2) | 44.8 (23.7) | 61.4 (39.9) | 0.24 |
|  | Mean duration of hospitalization during current phase (previous and current):  Days, median (IQR) | 36.0 (30.0-49.0) | 44.0 (30.0-60.0) | 60.0 (29.0-90.0) | 0.24 |
| **Ambulatory care** | Number of visits: follow-up, median (SD) | 16.9 (34.6) | 28.3 (37.3) | 34.1 (32.0) | 0.00* |
| **Treatment duration** | Treatment duration: weeks in early/intensive phase:  mean (SD) | 5.9 (4.4) | 5.8 (3.5) | 8.7 (6.8) | 0.39 |
|  | Treatment duration: weeks in late/continuation phase: mean (SD) | 7.9 (4.3) | 8.9 (5.9) | 25.9 (17.6) | 0.01* |
|  | Hours lost by the patient, mean (SD) | 32.6 (105.6) | 107.5 (154.0) | 85.0 (90.7) | 0.00* |
|  | Hours lost by guardian/caregiver, mean (SD) | 16.0 (12.7) | 6.3 (-) | 10.5 (5.6) | 0.68 |

BPaL = bedaquiline, linezolid & pretomanid; SSOR = standard short all-oral regimen; SLOR = standard long all-oral regimen; SD = standard deviation; IQR = interquartile range; DOTs = directly observed therapy; 95% CI = 95% confidence interval

^*^p≤0.05 using Kruskal-Wallis non-parametric test for continuous data

| **Supplementary Table 2. Unit costs and average quantities of ingredients used in DR-TB treatment by treatment group (N=112).** | | | | | | |
| --- | --- | --- | --- | --- | --- | --- |
|  |  |  | **Treatment group** | | |  |
|  |  |  | **BPaL (n=42)** | **SSOR (n=46)** | **SLOR (n=24)** |  |
|  | **Description** | **Unit cost (USD 2022)** | **Quantity (average)** | **Quantity (average)** | **Quantity (average)** | **Source** |
| **TB diagnostics/monitoring (variable costs)** | Smear Microscopy (average of Auramine + Ziehl-Neelsen) | $1,59 | 7,39 | 5,77 | 8,13 | South African National Health Laboratory Services (NHLS) State Price List (2018): Reference #31 |
|  | GeneXpert MTB/RIF | $13,80 | 0,00 | 0,28 | 0,42 |  |
|  | Line Probe Assay | $14,01 | 0,00 | 0,28 | 0,42 |  |
|  | Mycobacterium Growth Indicator Tube (average of growth + no growth) | $7,35 | 6,59 | 4,74 | 6,42 |  |
|  | Chest Radiography/X-ray | $17,97^†^ | 0,07 | 0,83 | 0,46 |  |
| **Client-facing provider interactions**  **(variable costs)** | Clinician (per minute) | $0,66 | 11,76 (38 minutes per interaction) | 15,70 (24 minutes per interaction) | 19,50 (21 minutes per interaction) | South African Department of Public Service and Administration (DPSA) Salary Scales (2019-2021): Reference #29 |
|  | Nurse (per minute) | $0,18 | 11,93 (13 minutes per interaction) | 15,36 (7,5 minutes per interaction) | 19,38 (9 minutes per interaction) |  |
|  | Counsellor (per minute) | $0,42 | 0,02 (25 minutes per interaction)^‡^ | 0,04 (25 minutes per interaction)^‡^ | 0,13 (25 minutes per interaction)^‡^ |  |
|  | Pharmacist (per minute) | $0,47 | 3,46 (12 minutes per interaction) | 2,38 (13 minutes per interaction) | 4,58 (12 minutes per interaction) |  |
|  | Psychologist (per minute) | $0,55 | 0,07 (38 minutes per interaction)^‡^ | 0,02 (38 minutes per interaction)^‡^ | 0,04 (38 minutes per interaction)^‡^ |  |
|  | Dietician (per minute) | $0,26 | 0,20 (17 minutes per interaction) | 0,57 (14 minutes per interaction) | 0,96 (13 minutes per interaction) |  |
|  | Social worker (per minute) | $0,28 | 3,05 (45 minutes per interaction)^‡^ | 0,77 (45 minutes per interaction)^‡^ | 0,71 (45 minutes per interaction)^‡^ |  |
|  | Audiologist (per minute) | $0,26 | 0,00 | 0,04 (38 minutes per interaction) | 0,00 |  |
| **TB drugs**  **(variable costs)** | Bedaquiline tablets (100mg) | $0,76 | 186,58 | 236,44 | 261,56 | South African National Department of Health (NDoH) Essential Medicine List and Master Procurement Catalogue (2021): Reference #30 |
|  | Levofloxacin break-line tablet (250mg) | $0,12 | 5,42 | 789,45 | 1006,93 |  |
|  | Linezolid tablet (600mg) | $3,13 | 183,39 | 102,04 | 119,04 |  |
|  | Pyrazinamide tablet (500mg) | $0,04 | 0,07 | 481,09 | 254,85 |  |
|  | Clofazimine tablets (100mg) | $0,54 | 2,07 | 205,36 | 289,42 |  |
|  | Terizidone capsule (250mg) | $0,54 | 1,80 | 0,00 | 651,15 |  |
|  | Pretomanid tablet (200mg) | $1,50^±^ | 169,54 | 3,35 | 0,00 |  |
|  | Delamanid tablet (50mg) | $1,81 | 3,13 | 41,02 | 407,01 |  |
|  | Ethionamide tablet (250mg) | $0,12 | 4,10 | 37,53 | 122,49 |  |
|  | Moxifloxacin tablet (400mg) | $0,37 | 0,00 | 3,57 | 0,00 |  |
|  | Ethambutol tablet (400mg) | $0,04 | 0,00 | 272,42 | 88,75 |  |
|  | Isoniazid tablet (100mg) | $0,03 | 0,44 | 938,97 | 289,52 |  |
| **Ancillary drugs**  **(variable costs)** | Hydrochlorothiazide tablet (12.5mg) | $0,01 | 5,80 | 1,19 | 0,00 |  |
|  | Enalapril tablet (10mg) | $0,01 | 4,10 | 0,00 | 0,00 |  |
|  | Amlodipine tablet (5mg) | $0,01 | 9,56 | 1,19 | 0,00 |  |
|  | Metformin tablet (500mg) | $0,01 | 4,78 | 0,00 | 0,00 |  |
|  | Paracetamol tablet (500mg) | $0,01 | 5,90 | 67,02 | 162,67 |  |
|  | Ibuprofen tablet (200mg) | $0,01 | 0,15 | 10,72 | 0,00 |  |
|  | Ceftriaxone injection (250mg) | $0,28 | 0,00 | 0,00 | 0,33 |  |
|  | Prednisone tablet (5mg) | $0,01 | 0,00 | 0,00 | 0,21 |  |
|  | Azithromycin tablet/capsule (250mg) | $0,22 | 0,00 | 0,00 | 0,42 |  |
|  | Aciclovir dispersible tablet (200mg) | $0,03 | 2,05 | 0,00 | 0,00 |  |
|  | Pyridoxine (Vitamin B6) tablet (25mg) | $0,01 | 161,56 | 691,96 | 1056,38 |  |
|  | Enoxaparin injection (40mg) | $2,56 | 0,00 | 0,60 | 0,00 |  |
|  | Metoclopramide tablet (10mg) | $0,01 | 0,00 | 13,74 | 17,54 |  |
|  | Fluconazole tablet/capsule (50mg) | $0,04 | 0,00 | 1,19 | 2,50 |  |
|  | Lansoprazole capsule (30mg) | $0,03 | 1,37 | 0,00 | 5,83 |  |
|  | Furosemide tablet (40mg) | $0,01 | 0,00 | 0,00 | 2,33 |  |
|  | Amoxicillin capsule (250mg) | $0,02 | 0,00 | 0,00 | 7,42 |  |
|  | Sulfamethoxazole and Trimethoprim tablet (400/80mg) | $0,01 | 0,00 | 22,30 | 0,83 |  |
|  | Folic acid tablet (5mg) | $0,01 | 7,17 | 21,09 | 223,42 |  |
|  | Metronidazole tablet (200mg) | $0,01 | 0,00 | 9,10 | 14,00 |  |
|  | Ferrous salt tablet/capsule (50-65mg) | $0,01 | 4,10 | 28,62 | 12,25 |  |
|  | Tramadol capsule/tablet (50mg) | $0,02 | 0,00 | 1,79 | 10,50 |  |
|  | Epilim tablet (100mg) | $0,07 | 0,00 | 28,00 | 239,92 |  |
|  | Omeprazole tablet/capsule (10mg) | $0,03 | 0,00 | 19,49 | 2,33 |  |
|  | Pregabalin capsule (25mg) | $0,08 | 0,00 | 74,47 | 0,00 |  |
|  | Magnesium chloride slow release tablet (500-700mg) | $0,03 | 0,00 | 2,38 | 1,17 |  |
|  | Potassium chloride tablet (600mg) | $0,05 | 0,00 | 53,62 | 0,00 |  |
|  | Amitriptyline tablet (10mg) | $0,02 | 0,00 | 8,94 | 0,00 |  |
|  | Lamotrigine dispersible tablet (5mg) | $0,12 | 0,00 | 23,83 | 0,00 |  |
|  | Loperamide tablet (2mg) | $0,02 | 0,00 | 0,00 | 1,17 |  |
|  | Baclofen tablet (10mg) | $0,04 | 0,00 | 1,19 | 0,00 |  |
|  | Pholcodine linctus (100ml) | $1,85 | 1,37 | 0,00 | 0,00 |  |
|  | Clotrimazole cream 1% (20g) | $0,26 | 0,00 | 0,60 | 0,00 |  |
|  | Bimatoprost eye drop (3ml) | $2,17 | 0,00 | 1,79 | 0,00 |  |
|  | Calcium gluconate injection 10% (10ml) | $0,52 | 0,00 | 5,77 | 1,17 |  |
|  | Levothyroxine sodium tablet (0.025mg) | $0,01 | 0,00 | 23,23 | 56,75 |  |
|  | Haloperidol capsule (0.5mg) | $0,10 | 0,00 | 0,00 | 35,00 |  |
|  | Dorzolamide and Timolol ophthalmic drops (20 and 5mg/ml) | $3,09 | 0,00 | 1,19 | 0,00 |  |
| **Laboratory tests (variable costs)** | HIV Rapid Screen Test | $3,17 | 0,02 | 0,00 | 0,04 | South African National Health Laboratory Services (NHLS) State Price List (2018): Reference #31 |
|  | HIV Serology | $3,97 | 0,05 | 0,00 | 0,00 |  |
|  | Glucose (blood) | $2,18 | 0,02 | 0,23 | 0,33 |  |
|  | Glucose (urine) | $2,18 | 0,07 | 0,30 | 0,46 |  |
|  | Urea | $2,18 | 0,02 | 0,00 | 0,00 |  |
|  | Human Chorionic Gonadotropin | $6,10 | 0,00 | 0,04 | 0,00 |  |
|  | Alanine Transaminase | $3,27 | 0,27 | 0,36 | 0,04 |  |
|  | Haemoglobin | $1,30 | 1,63 | 0,87 | 1,75 |  |
|  | COVID-19 Polymerase Chain Reaction | $40,94 | 0,02 | 0,11 | 0,29 |  |
|  | Alkaline Phosphatase | $3,11 | 0,20 | 0,13 | 0,04 |  |
|  | Gamma-glutamyl Transferase | $3,27 | 0,22 | 0,11 | 0,00 |  |
|  | Creatinine | $2,18 | 0,27 | 0,21 | 0,38 |  |
|  | Blood Urea Nitrogen | $2,18 | 0,02 | 0,06 | 0,42 |  |
|  | Prothrombin Index | $3,40 | 0,00 | 0,04 | 0,00 |  |
|  | Albumin | $2,89 | 0,15 | 0,09 | 0,00 |  |
|  | Protein (total) | $1,87 | 0,17 | 0,09 | 0,00 |  |
|  | Thyroid Stimulating Hormone | $12,58 | 0,00 | 0,06 | 0,00 |  |
|  | Bilirubin (total) | $2,54 | 0,15 | 0,06 | 0,00 |  |
|  | Calcium | $2,18 | 0,00 | 0,00 | 0,04 |  |
|  | Full Blood Count | $4,17 | 6,32 | 3,64 | 6,79 |  |
|  | Full Live Function Panel | $28,80 | 0,27 | 0,32 | 0,25 |  |
|  | Sodium | $2,18 | 0,05 | 0,00 | 0,13 |  |
|  | Potassium | $2,18 | 0,95 | 0,00 | 0,13 |  |
|  | Chloride (sweat/urine) | $1,56 | 0,00 | 0,00 | 0,04 |  |
|  | Bicarbonate | $3,05 | 0,00 | 0,00 | 0,13 |  |
|  | Differential Count | $2,29 | 0,15 | 0,09 | 0,00 |  |
| **Overhead**  **(fixed-costs)** | Rent | - | $466,34^¶^ | $475,65^§^ | $742,04^¥^ | Financial records and reports from a single centralized government public TB hospital and specialized referral center located in Johannesburg, South Africa (2021) |
|  | Security | - | $628,84^¶^ | $641,40^§^ | $1 000,62^¥^ |  |
|  | Transportation | - | $32,12^¶^ | $32,76^§^ | $51,11^¥^ |  |
|  | Telecommunications | - | $509,66^¶^ | $519,83^§^ | $810,98^¥^ |  |
|  | Food and supplies | - | $2,04^¶^ | $2,08^§^ | $3,24^¥^ |  |
|  | Fuel (chemicals, oil, gas, wood, coal) | - | $13,33^¶^ | $13,59^§^ | $21,21^¥^ |  |
|  | Fumigation | - | $378,69^¶^ | $386,25^§^ | $602,58^¥^ |  |
|  | Medical waste | - | $101,38^¶^ | $103,40^§^ | $161,31^¥^ |  |
|  | Medical supplies (gloves etc) | - | $49,51^¶^ | $50,50^§^ | $78,78^¥^ |  |
|  | Maintenance and repairs | - | $215,05^¶^ | $219,34^§^ | $342,19^¥^ |  |
|  | Office supplies and stationary | - | $387,44^¶^ | $394,60^§^ | $620,11^¥^ |  |
|  | Cleaning supplies and materials | - | $7,09^¶^ | $7,22^§^ | $11,35^¥^ |  |
|  | Linen | - | $30,41^¶^ | $33,12^§^ | $35,23^¥^ |  |
| **Equipment (fixed-costs)** | Equipment (various) | - | $150,66^¶^ | $153,66^§^ | $239,73^¥^ | Various South African online retailers |

DR = drug-resistant; TB = tuberculosis; BPaL = bedaquiline, pretomanid & linezolid; SSOR = standard short all-oral regimen; SLOR = standard long all-oral regimen; USD = United States Dollar; MTB/RIF = mycobacterium tuberculosis/rifampicin; mg = milligram; ml = millilitre; g = gram

^†^Unit cost sourced from personal communication with radiologist at large tertiary-level academic hospital in Johannesburg, South Africa

^‡^Consistent interaction/consultation duration (minutes per interaction) applied across treatment groups due to limited observable interactions

^±^Due to pretomanid being unavailable on tender in South Africa at the time of the analysis (pretomanid donated to South Africa’s National TB Programme), unit price for pretomanid was sourced via personal communication with TB Alliance in June 2023

Unit cost sourced from unpublished South African National Health Laboratory Services (NHLS) price list (2023/2024)

^¶^Total cost per patient episode (all outcomes) based on 41 in-patient days and 6.9 out-patient visits

^§^Total cost per patient episode (all outcomes) based on 42 in-patient days and 7.5 out-patient visits

^¥^Total cost per patient episode (all outcomes) based on 66 in-patient days and 8.0 out-patient visits
